# Evaluating the Glycemic Effects of Dolutegravir and Its Predictors Among People With Human Immunodeficiency Virus in Uganda: A Prospective Cohort Study

**DOI:** 10.1093/ofid/ofae596

**Published:** 2024-10-08

**Authors:** Collins Ankunda, Curthbert Agolor, Yvonne Karamagi, Susan Nakubulwa, Sharon Namasambi, Ivan Kasamba, Semei Mukama Christopher, Patience Kukundakwe, Mary Odiit, Ivan Mubangizi, Jude Emunyu, Diana Nakitto Kesi, Victoria Nambasa, Helen Byomire Ndagije, Barbara Mukasa

**Affiliations:** Mildmay Research Centre Uganda, Mildmay Uganda, Kampala, Uganda; Mildmay Research Centre Uganda, Mildmay Uganda, Kampala, Uganda; Mildmay Research Centre Uganda, Mildmay Uganda, Kampala, Uganda; Mildmay Research Centre Uganda, Mildmay Uganda, Kampala, Uganda; Mildmay Research Centre Uganda, Mildmay Uganda, Kampala, Uganda; Mildmay Research Centre Uganda, Mildmay Uganda, Kampala, Uganda; Mildmay Research Centre Uganda, Mildmay Uganda, Kampala, Uganda; Mildmay Research Centre Uganda, Mildmay Uganda, Kampala, Uganda; Mildmay Research Centre Uganda, Mildmay Uganda, Kampala, Uganda; Mildmay Research Centre Uganda, Mildmay Uganda, Kampala, Uganda; Mildmay Research Centre Uganda, Mildmay Uganda, Kampala, Uganda; Directorate of Product Safety, National Drug Authority, Kampala, Uganda; Directorate of Product Safety, National Drug Authority, Kampala, Uganda; Directorate of Product Safety, National Drug Authority, Kampala, Uganda; Mildmay Research Centre Uganda, Mildmay Uganda, Kampala, Uganda

**Keywords:** antiretroviral therapy, diabetes, dolutegravir, HIV, hyperglycemia

## Abstract

**Background:**

Dolutegravir (DTG), a key component of the recommended human immunodeficiency virus (HIV) treatment regimens in Uganda, has been associated with hyperglycemia. We evaluated its influence on hyperglycemia risk to create a hyperglycemia risk stratification tool for patient monitoring.

**Methods:**

We conducted a prospective cohort study at 3 sites with 628 HIV-positive patients on tenofovir disoproxil fumarate, lamivudine, and dolutegravir (TLD). Participants included both nucleoside reverse transcriptase inhibitor–experienced (exposed) and antiretroviral therapy (ART)–naive (nonexposed) groups. Follow-up occurred every 6 months with random blood sugar (RBS) testing every 3 months. Participants with RBS ≥7 mmol/L were classified as hyperglycemic and underwent glycated hemoglobin (HbA1c) testing, confirming diabetes with a 6.5% cut-off.

**Results:**

The study found a hyperglycemia incidence rate of 24.5 (95% confidence interval [CI], 19.3–31.1) cases per 100 person-years (PY) and a diabetes incidence rate of 5.8 cases (95% CI, 3.6–9.3) per 100 PY. Hyperglycemia incidence was slightly lower in nonexposed (20.8 cases per 100 PY) versus exposed groups (25.2 cases per 100 PY). Multivariable analysis indicated a trend toward lower hyperglycemia risk (adjusted hazard ratio [aHR], 0.78 [95% CI, .37–1.66]; *P* = .52) and substantially lower diabetes incidence (aHR, 0.34 [95% CI, .04–2.82]; *P* = .32) in the nonexposed group. Significant factors for hyperglycemia included age (*P* < .001), study site (*P* < .001), and DTG-based ART duration (*P* = .02).

**Conclusions:**

Our study showed an increased incidence of hyperglycemia with age, study site, and duration of DTG exposure in people with HIV on TLD. We suggest integrated screening and care for hyperglycemia and diabetes in HIV services, especially when initiating DTG regimens.

Diabetes mellitus (DM) is a leading cause of death in adults globally [1]. Prolonged hyperglycemia signals early diabetes risk, crucial for monitoring, especially in predisposed individuals [2]. In 2019, global diabetes prevalence was 9.3% (20–79 years) and 19.9% (65–79 years), with Uganda's 2014 survey at 1.4% [1]. The DM–human immunodeficiency virus (HIV) treatment association in sub-Saharan Africa is understudied [3]. Antiretroviral therapy (ART) use may influence DM development, complicating HIV/AIDS management [4, 5]. Uganda reports symptomatic hyperglycemia with DTG regimens, but data on incidence are limited [6, 7]. Dolutegravir (DTG) is believed to chelate and reduce serum magnesium levels, potentially affecting glucose transport via GLUT-4 receptor, leading to increased liver glucose production [8, 9].

Uganda's HIV guidelines recommend DTG with tenofovir disoproxil fumarate, lamivudine, and dolutegravir (TLD) as the preferred first-line regimen for people with HIV (PLHIV), aligning with global trends [10, 11]. Prioritizing new HIV diagnoses and treatment readiness, TLD is chosen for its high resistance barrier [10, 12–14]. However, DTG regimens may present hyperglycemia risks, as observed in studies like Second-line Protease Inhibitor-based Regimens to Integrase Inhibitor-based Regimens in Treatment-Naïve Patients (SPRING-2) and Study of Integrase Inhibitor and Nucleoside Reverse Transcriptase Inhibitors in a Once-Daily Regimen (SINGLE). There have been reports of grade 2 (6.95–13.88 mmol/L) and grade 3 (blood glucose >13.88 mmol/L) hyperglycemia associated with DTG use, though the extent of these findings remains unclear [15–17]. Despite widespread DTG adoption, concerns about hyperglycemia and other adverse effects persist [18].

Despite DTG's efficacy in HIV treatment, limited hyperglycemia data in Uganda hinders risk assessment. This study aims to address this gap by systematically assessing hyperglycemia incidence among PLHIV on DTG-based regimens. The World Health Organization (WHO) underscores the importance of staying informed about drug risks and encourages integrating emerging evidence into clinical guidelines [19].

Therefore, this study addresses a critical knowledge gap and aligns with WHO recommendations, contributing to evidence-based decision-making in HIV care. The study evaluated the association between prior exposure to nucleoside reverse transcriptase inhibitors (NRTIs) and the risk of hyperglycemia or DM in order to guide risk stratification and tailored interventions, ultimately enhancing the quality of HIV care and treatment in Uganda and beyond.

## MATERIALS AND METHODS

### Study Design

This prospective cohort study evaluated the incidence of hyperglycemia among adults receiving DTG as part of their ART regimen. The study spanned 14 months, from July 2021 to September 2022. Participants were enrolled and followed for 6 months, with clinical and laboratory assessments every 3 months to evaluate hyperglycemia related to ART history.

### Study Sites and Study Population

Electronic lists from Mildmay Uganda Hospital (site 1), Luwero Hospital (site 2), and Nyimbwa Health Centre IV (site 3) ART clinics were used to sample participants. Cluster sampling organized the lists into study groups, followed by random sampling. The exposed group comprised participants on TLD who had prior exposure to NRTIs and nonnucleoside reverse transcriptase inhibitors (NNRTIs), while the exposed group included ART-naive participants on TLD, representing their first ART regimen. Adults on TLD with undetectable viral loads and no history of diabetes were included. Inclusion criteria required consent and an adult >18 years of age, while exclusion criteria included history of hyperglycemia, detectable HIV viremia, unclear ART history, and pregnancy. A proportionate sample size was selected to ensure representation across different settings: urban, periurban, and rural.

### Sample Size Calculation

The study opted to use data for protease inhibitors due to lack data on integrase strand inhibitors. Past research indicated protease inhibitors to be independently associated with hyperglycemia (incidence rate ratio, 5 [95% confidence interval {CI}, 1.3–19.4]) [20]. The sample size for this study (N = 628) was determined using Open Epi software to achieve 80% power, considering potential follow-up losses with 2 groups in the study and 2-sided 95% CI.

### Data Collection and Management

Data on participants' sociodemographic characteristics and medical history and examination were collected using a combination of participant interviews and abstraction of data from ART registers, patient cards, and electronic medical records. All data were entered into the Open Data Kit platform for electronic storage and analysis. To ensure data accuracy, regular checks were conducted against hard copies of study materials.

### Laboratory Procedures and Interpretation of Laboratory Results

At each 3-month study visit, participants underwent random blood sugar (RBS) testing using an On Call Plus glucometer, chosen for its cost-effectiveness and lack of dietary restrictions. Participants with RBS levels ≥7 mmol/L had additional glycated hemoglobin (HbA1c) testing to confirm diabetes, using a 6.5% HbA1c cut-off [21]. HbA1c analysis was conducted with the AFI 6000 Fluorescence Immuno Assay Analyzer or Cobas C311 at the ISO 15189 certified Mildmay Uganda Hospital laboratory. The 7 mmol/L hyperglycemia cut-off is supported by Bowen et al, who found elevated RBS levels (≥5.6 mmol/L) to be strongly linked to undiagnosed diabetes (odds ratio [OR], 31.2), persisting after adjustment (OR, 20.4) [22]. Diabetes likelihood increased with higher RBS: 100–119 mg/dL (5.6–6.6 mmol/L) (OR, 7.1); 120–139 mg/dL (6.7–7.7 mmol/L) (OR, 30.3); and ≥140 mg/dL (≥7.8 mmol/L) (OR, 256). Additionally, the Division of AIDS grading system classifies high nonfasting blood glucose levels from 6.44 to <8.89 mmol/L as mild [23].

### Data Analysis

Data analysis was performed using Stata version 15 software. Descriptive statistics were used to summarize participant characteristics, and Cox regression models were employed to assess the association between variables. Multivariable models were adjusted for potential confounders.

### Ethical Considerations

Ethical approval was obtained from the Mildmay Uganda Research Ethics Committee (#REC REF 0812-2020) and the Uganda National Council for Science and Technology (HS1273ES). Participants were treated with confidentiality, and written informed consent was obtained prior to enrollment in the study.

## RESULTS

The majority of the participants were female (n = 419 [67%]), aged ≥40 years (n = 327 [52%]), non–alcohol users (n = 453 [72%]), nonsmokers (n = 601 [96%]), of normal body mass index (BMI) (n = 347 [56%]), of normal waist circumference (n = 322 [52%]), of normal waist-to-hip ratio (n = 367 [59%]), normotensive (n = 386 [63%]), previously exposed to tenofovir disoproxil fumarate NRTI backbone ART regimen (n = 407 [65%]), ART experienced for ≥5 years (n = 404 [64%]), euglycemic at baseline (n = 605 [96%]), and asymptomatic (n = 366 [58%]), as shown in Table 1.

**Table 1.** Baseline Characteristics of Study Participants

Category	No. (%) (N = 628)
Study site	
Site 1	390 (62.1)
Site 2	188 (29.9)
Site 3	50 (8.0)
Sex	
Female	419 (67)
Male	209 (33)
Age, y	
18–29	124 (19.7)
30–39	177 (28.2)
≥40	327 (52.1)
Marital status	
Single	312 (49.7)
Married	221 (35.2)
Separated/divorced/widowed	95 (15.1)
Alcohol intake	
No	453 (72.2)
Weekly or occasionally	162 (25.8)
Daily	12 (1.9)
Smoking status	
Yes	26 (4.1)
No	601 (95.9)
BMI, kg/m^2^	
≤24.9 (normal)	347 (56.1)
25–29.9 (overweight)	168 (27.1)
≥30 (obesity)	104 (16.8)
Waist circumference	
Abnormal: ≥94 cm (men); ≥80 cm (women)	301 (48.3)
Normal: <94 cm (men); <80 cm (women)	322 (51.7)
Waist-to-hip ratio	
Abnormal: >0.95 (men); >0.85 (women)	252 (40.7)
Normal: <0.95 (men); <0.85 (women)	367 (59.3)
Hypertension level (systolic/diastolic, mm Hg)	
Normal (110–129/70–79)	386 (62.5)
Mild (130–139/80–89)	188 (30.4)
Grade 2/3 hypertension (≥140/≥90)	44 (7.1)
ART regimen exposure	
TDF NRTI backbone	407 (64.8)
Zidovudine NRTI backbone	121 (19.3)
Abacavir NRTI backbone	4 (0.6)
TLD at initiation	96 (15.3)
Overall duration on ART	
<2 y	76 (12.1)
2 to <5 y	148 (23.6)
≥5 y	404 (64.3)
Duration on current ART	
<1 y	172 (27.4)
1 to <2 y	240 (38.2)
≥2 y	216 (34.4)
Random blood glucose	
<7.0 mmol/L	605 (96.3)
≥7.0 mmol/L	23 (3.7)

Abbreviations: ART, antiretroviral therapy; BMI, body mass index; NRTI, nucleoside reverse transcriptase inhibitor; TDF, tenofovir disoproxil fumarate; TLD, tenofovir disoproxil fumarate, lamivudine, and dolutegravir.

### Incidence of Hyperglycemia

The study found an overall hyperglycemia incidence of 24.5 per 100 person-years (PY). The incidence was higher in the exposed group (25.2 per 100 PY) compared to the nonexposed group (20.8 per 100 PY) (hazard ratio [HR], 0.74 [95% CI, .36–1.50]; *P* = .38). Hyperglycemia risk was associated with increasing age, marital status, study site, and duration on TLD. However, the increased risk of hyperglycemia was linked to the duration of DTG exposure and not the overall duration on ART (Table 2).

**Table 2.** Incidence of Hyperglycemia and Relative Risks

Characteristic	No. with Hyperglycemia/PY	Incidence of Hyperglycemia (95% CI)	Hazard Ratio (95% CI)	*P* Value
All respondents	68/277.4	24.5 (19.3–31.1)	…	
Previous exposure to DTG				.38
Exposed	59/234.0	25.2 (19.5–32.5)	1	
Nonexposed	9/43.4	20.8 (10.8–39.9)	0.74 (.36–1.50)	
ART regimen prior to the current regimen				.58
TDF NRTI backbone	45/178.0	25.3 (18.9–33.9)	1	
ZDV NRTI backbone	14/54.4	25.8 (15.3–43.5)	1.03 (.56–1.90)	
ABC NRTI backbone	0/1.7	…	…	
TLD at initiation	9/43.4	20.8 (10.8–39.9)	0.74 (.36–1.52)	
Study site				<.001
Site 1	28/176.2	15.9 (11.0–23.0)	1	
Site 2	37/78.8	46.9 (34.0–64.8)	2.71 (1.58–4.65)	
Site 3	3/22.3	13.4 (4.3–41.6)	0.66 (.19–2.24)	
Sex of respondent				.96
Male	26/90.5	28.7 (19.6–42.2)	1	
Female	42/186.9	22.5 (16.6–30.4)	1.01 (.59–1.73)	
Age, y				<.001
18–29	5/57.1	8.8 (3.6–21.0)	1	
30–39	14/81.1	17.3 (10.2–29.2)	1.90 (.68–5.29)	
≥40	49/139.2	35.2 (26.6–46.6)	3.56 (1.39–9.09)	
Marital status				.04
Single	23/139.3	16.5 (11.0–24.9)	1	
Married	29/97.1	29.9 (20.8–43.0)	1.78 (1.02–3.09)	
Separated/divorced/widowed	16/40.5	39.5 (24.2–64.5)	2.09 (1.09–4.01)	
Alcohol intake				.33
No	45/199.2	22.6 (16.9–30.3)	1	
Weekly or occasionally	20/72.9	27.4 (17.7–42.5)	1.15 (.68–1.95)	
Daily	3/4.8	63.0 (20.3–195.3)	2.01 (.61–6.63)	
Smoking status				.30
No	63/265.0	23.8 (18.6–30.4)	1	
Yes	5/11.9	42.1 (17.5–101.1)	1.63 (.65–4.10)	
BMI, kg/m^2^				.77
≤24.9	40/152.1	26.3 (19.3–35.9)	1	
25–29.9	16/74.5	21.5 (13.2–35.1)	0.85 (.48–1.53)	
≥30	12/47.0	25.5 (14.5–45.0)	1.11 (.58–2.14)	
Abnormal waist circumference				.50
≥94 cm (men)	34/140.4	24.2 (17.3–33.9)	1	
≥80 cm (women)	34/132.8	25.6 (18.3–35.8)	1.19 (.73–1.94)	
Abnormal waist-hip ratio				.93
>0.95 (men)	41/159.2	25.7 (19.0–35.0)	1	
>0.85 (women)	27/111.9	24.1 (16.5–35.2)	1.02 (.62–1.68)	
Hypertension level				.34
Normal	41/169.2	24.2 (17.8–32.9)	1	
Mild	19/82.5	23.0 (14.7–36.1)	0.97 (.56–1.67)	
Grade 2/3 hypertension	8/19.0	42.2 (21.1–84.3)	1.75 (.81–3.75)	
Overall duration on ART				.55
<2 y	6/34.1	17.6 (7.9–39.1)	1	
2 to <5 y	17/65.4	26.0 (16.2–41.8)	1.45 (.55–3.79)	
≥5 y	45/177.3	25.4 (18.9–34.0)	1.58 (.66–3.79)	
Duration on TLD				.01
<1 y	15/77.5	19.3 (11.7–32.1)	1	
1 to <2 y	19/107.9	17.6 (11.2–27.6)	1.69 (.67–4.27)	
≥2 y	34/91.4	37.2 (26.6–52.0)	6.09 (1.80–20.61)	

Abbreviations: ABC, abacavir; ART, antiretroviral therapy; BMI, body mass index; CI, confidence interval; DTG, dolutegravir; NRTI, nucleoside reverse transcriptase inhibitor; PY, person-years; TDF, tenofovir disoproxil fumarate; TLD, tenofovir disoproxil fumarate, lamivudine, and dolutegravir; ZDV, zidovudine.

### Association Between Previous NRTI Exposure and the Incidence of Hyperglycemia Among Patients on TLD

The multivariable analysis showed lower hyperglycemia risk in the nonexposed group, but this was not statistically significant (adjusted HR [aHR], 0.78 [95% CI, .37–1.66]; *P* = .52). Influencing factors included study site, age, marital status, and alcohol consumption, but not BMI. The relationship was modified by alcohol (interaction *P* = .09) and sex (interaction *P* = .14), with nondrinkers and women showing lower incidence rates (22.5 [95% CI, 16.6–30.4]) compared to their counterparts (28.7 [95% CI, 19.6–42.2]).

### Incidence of Diabetes Mellitus

The study found a DM incidence of 5.8 per 100 PY, higher in the exposed group (6.5 per 100 PY) than the nonexposed group (2.2 per 100 PY). Incidence varied by DTG duration, age, marital status, alcohol, smoking, BMI, waist measures, hypertension, and study site (Table 3).

**Table 3.** Incidence of Diabetes Mellitus and Relative Risks

Characteristic	No. With Diabetes Mellitus/PY	Incidence of Diabetes per 100 PY (95% CI)	Hazard Ratio (95% CI)	*P* Value
All participants	17/292.6	5.8 (3.6–9.3)	…	
Previous exposure to ART				.17
Exposed	16/246.5	6.5 (4.0–10.6)	1	
Nonexposed	1/46.1	2.2 (.3–15.4)	0.31 (.04–2.33)	
ART backbone among the exposed				.54
TDF NRTI backbone	12/188.3	6.4 (3.6–11.2)	1	
ZDV NRTI backbone	4/56.5	7.1 (2.7–18.9)	0.95 (.30–3.04)	
ABC NRTI backbone	0/1.7	…	…	
TLD at initiation	1/46.1	2.2 (.3–15.4)	0.30 (.04–2.33)	
Study site				.03
Site 1	9/181.8	5.0 (2.6–9.5)	1	
Site 2	8/87.2	9.2 (4.6–18.3)	1.98 (.68–5.76)	
Site 3	0/23.6	…	…	
Sex of respondent				.77
Male	6/96.8	6.2 (2.8–13.8)	1	
Female	11/195.8	5.6 (3.1–10.1)	1.17 (.40–3.40)	
Age, y				.16
18–29	1/57.9	1.7 (.2–12.3)	1	
30–39	4/83.8	4.8 (1.8–12.7)	2.81 (.31–25.28)	
≥40	12/150.8	8.0 (4.5–14.0)	4.08 (.51–32.36)	
Marital status				.11
Single	6/144.0	4.2 (1.9–9.3)	1	
Married	5/105.0	4.8 (2.0–11.4)	1.08 (.33–3.59)	
Separated/divorced/widowed	6/43.1	13.9 (6.3–31.0)	3.41 (1.05–11.04)	
Alcohol intake				.14
No	15/207.6	7.2 (4.4–12.0)	1	
Weekly or occasionally	2/78.5	2.5 (.6–10.2)	0.32 (.07–1.38)	
Daily	0/6.0		…	
Smoking status				.94
No	16/278.6	5.7 (3.5–9.4)	1	
Yes	1/13.5	7.4 (1.0–52.6)	1.08 (.14–8.23)	
BMI, kg/m^2^				.31
≤24.9	7/161.6	4.3 (2.1–9.1)	1	
25–29.9	5/78.6	6.4 (2.6–15.3)	1.49 (.47–4.72)	
≥30	5/48.6	10.3 (4.3–24.7)	2.55 (.80–8.20)	
Abnormal waist circumference				.07
≥94 cm (men)	5/149.0	3.4 (1.4–8.1)	1	
≥80 cm (women)	12/139.4	8.6 (4.9–15.2)	2.68 (.93–7.73)	
Abnormal waist-hip ratio				.11
>0.95 (men)	7/169.5	4.1 (2.0–8.7)	1	
>0.85 (women)	10/117.4	8.5 (4.6–15.8)	2.24 (.84–6.00)	
Hypertension level				.78
Normal	10/179.2	5.6 (3.0–10.4)	1	
Mild	5/86.2	5.8 (2.4–13.9)	1.04 (.35–3.05)	
Grade 2/3 hypertension	2/20.5	9.7 (2.4–39.0)	1.78 (.38–8.27)	
Overall duration on ART				.75
<2 y	1/35.8	2.8 (.4–19.8)	1	
2 to <5 y	5/69.3	7.2 (3.0–17.3)	2.21 (.24–20.60)	
≥5 y	11/187.0	5.9 (3.3–10.6)	1.93 (.24–15.79)	
Duration on TLD				.03
<1 y	2/81.1	2.5 (.6–9.9)	1	
1 to <2 y	6/111.2	5.4 (2.4–12.0)	12.79 (1.57–103.8)	
≥2 y	9/99.8	9.0 (4.7–17.3)	20.09 (1.08–372.3)	

Abbreviations: ABC, abacavir; ART, antiretroviral therapy; BMI, body mass index; CI, confidence interval; DTG, dolutegravir; NRTI, nucleoside reverse transcriptase inhibitor; PY, person-years; TDF, tenofovir disoproxil fumarate; TLD, tenofovir disoproxil fumarate, lamivudine, and dolutegravir; ZDV, zidovudine.

### Flowchart for Random Blood Glucose and HbA1c

**Figure 1.**
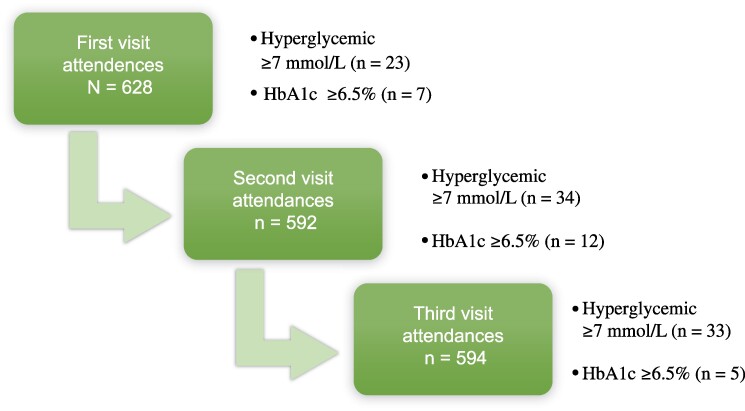
Study flowchart showing test results for random blood sugar (RBS) and glycated hemoglobin (HbA1c).

In the study, 90 hyperglycemic RBS test results were identified, with 17 cases classified as diabetic among 68 unique participants. Among these, 23 RBS results ≥7 mmol/L were found during the first visit, 34 during the second, and 33 during the third. Of these, 7, 12, and 5, respectively, were classified as diabetic during the respective visits (Figure 1).

### Association Between Previous ART Exposure and Incidence of Diabetes

After adjusting for potential confounders, the study found that being in a nonexposed group was associated with a 66% lower incidence of diabetes compared to the exposed group (aHR, 0.34 [95% CI, .04–2.82]; *P* = .32). The association between previous ART exposure and diabetes incidence was confounded by various factors, including study site, duration on DTG-based ART, age, and marital status.

### Predictors of Hyperglycemia Among Patients on TLD

Factors associated with hyperglycemia included age (*P* < .001), study site (*P* < .001), and duration of DTG-based ART (*P* = .02). Hyperglycemia incidence was higher among those older, married, and with prolonged ART. Site 2 participants had a 3.5 times higher incidence (Table 4).

**Table 4.** Factors Associated With the Incidence of Hyperglycemia

Category	Hazard Ratio (95% CI)	*P* Value
Study site		
Site 1	1	<.001
Site 2	3.48 (1.96–6.18)	
Site 3	0.66 (.19–2.25)	
Age, y		
18–29	1	<.001
30–39	1.75 (.62–4.93)	
≥40	4.61 (1.76–12.08)	
Marital status		
Single	1	.062
Married	1.99 (1.12–3.52)	
Separated/divorced/widowed	1.47 (.75–2.87)	
Duration on current ART (TLD)		
<1 y	1	.024
1 to <2 y	1.40 (.57–3.46)	
≥2 y	5.74 (1.54–21.20)	

Abbreviations: ART, antiretroviral therapy; CI, confidence interval; TLD, tenofovir disoproxil fumarate, lamivudine, and dolutegravir.

## DISCUSSION

In Uganda, the main considerations for ART initiation include a new diagnosis of HIV infection and patients’ readiness to start ART. The standard initial regimen should be TLD unless any of the components of this regimen are contraindicated. This is principally because DTG offers a higher genetic barrier to drug resistance as compared to NNRTIs and has not yet been extensively used previously in this setting [13]. However, alongside the benefits of this preferred regimen, come challenges with adverse effects, among which the risk of hyperglycemia is highest [18].

This multisite prospective cohort study observed 68 hyperglycemia events (11%) among 628 participants over 6 months, with an incidence rate of 24.5 per 100 PY. This aligns with the SAILING (Safety and Efficacy of Dolutegravir in Subjects Failing Integrase Inhibitor-Based Regimen) trial, which reported hyperglycemia-related laboratory abnormalities in 10% of participants, both with similar follow-up periods and ART-experienced participants [17].

However, these results contrast with the SPRING-2 and SINGLE studies, which reported lower hyperglycemia incidence rates of 7% (26/411) and 8% (34/414), respectively [15, 16]. This difference could be attributed to several factors such as the study design, participant selection criteria, and study setting. Our study employed an observational design reflecting real-world data and clinical practice, where ART prescription follows public health approach and pretreatment comorbidity screening is often more clinical than laboratory based. In contrast, the SPRING-2 and SINGLE studies were rigorous clinical trials with strict eligibility criteria [15, 16]. This difference in participant selection could have influenced the observed hyperglycemia rates.

In a 6-month period, this study observed an overall diabetes incidence of 5.8 cases (95% CI, 3.6–9.3) per 100 PY, which is comparable to 4.7 cases per 100 PY reported in a 4-year follow-up in the Multicenter AIDS Cohort Study (MACS) among HIV-infected men receiving ART [24]. Our study captured more incident diabetes cases than MACS population at 4 years; therefore, extending the follow-up period in future studies could potentially yield even higher DM incidence rates. Despite DM being an exclusion criterion at enrollment, the study identified 23 participants with hyperglycemia and 7 with diagnosed diabetes who were unaware of their condition (Figure 1). These findings are consistent with a study carried out in Tanzania, where 95% of HIV-infected adults with diabetes were not aware of their diagnosis [25]. Similarly, low DM awareness rates have been reported in other African studies [25].

Our study revealed an increased risk of hyperglycemia with increasing age. Participants aged ≥40 years had a 4.6-fold increased risk of hyperglycemia compared to those aged 18–29 years (aHR, 4.6 [95% CI, 1.8–12.1]). This age-related risk was further amplified by factors such as obesity, daily alcohol consumption, abnormal waist-to-hip ratio, and moderate/severe hypertension. These findings align with previous research demonstrating that increasing age, along with associated genetic changes and lifestyle factors, can contribute to a higher risk of hyperglycemia [26]. This observation suggests that initiating DTG-based regimens in older adults might present a double burden: the risk of HIV and the potential increased risk of hyperglycemia. Consequently, it highlights the importance of vigorous hyperglycemia screening for HIV-infected individuals within these high-risk groups before starting DTG therapy.

The duration on DTG-based regimen was one of the factors that we found to be predictive of hyperglycemia. Compared to DTG-based ART for <1 year, a duration of at least 2 years was associated with an increased incidence of hyperglycemia (aHR, 5.7 [95% CI, 1.5–21.2]). These findings are similar to those in a study by Namara et al, where PLHIV who had previously been prescribed a DTG-based regimen had 7 times greater odds of having hyperglycemia [26]. This similar trend has been suggested in the pivotal DTG trials, which indicate an increased risk of hyperglycemia with extended exposure to DTG combination treatment as well as treatment duration [17].

Uniquely, study site 2 (Luwero Hospital) had a notable association with hyperglycemia compared to Mildmay Uganda Hospital (site 1) and at Nyimbwa Health Centre IV (site 3). Specifically, 20% of participants at site 2 developed hyperglycemia, with an incidence rate of 46.9 per 100 PY (95% CI, 34.0–64.8). This was significantly higher than the rates at site 3, where 6% experienced hyperglycemia (13.4 per 100 PY, 95% CI, 4.3–41.6), and site 1, with 7.2% affected (15.9 per 100 PY, 95% CI, 11.0–23.0). Participants from site 2 had a threefold higher risk of developing hyperglycemia compared to those from site 1, with an aHR of 3.5 (95% CI, 2.0–6.2).

However, there have not been many studies to ascertain how a study setting could influence the incidence of hyperglycemia. Such an observation could be attributed to the fact that study site 2 is at the hospital level with a much wider catchment area in terms of access to ART services compared to its counterpart in the same rural setting (site 3, Nyimbwa Health Centre IV). The diversity in study participant composition could have clouded the true reflection of findings at site 2. Despite having almost the same catchment area and participant composition, the same phenomenon was not observed at site 1.

This could be explained by the difference in the study participants' composition considering that site 1 is in a periurban setting where access to screening services and health information is improved compared to a rural setting. Therefore, this would warrant a study to understand the site-related factors that could have contributed to these findings.

The study found no significant association between prior NRTI exposure and hyperglycemia among TLD users (*P* = .52), with a wide CI indicating insufficient power. Age, alcohol use, waist circumference, marital status, and DTG-based ART duration (but not overall ART duration) were confounders. Enhanced hyperglycemia screening for older adults and long-term DTG users is recommended, alongside tailored public health strategies.

Our study's prospective cohort design allowed for precise monitoring of newly occurring events and clear differentiation between hyperglycemia and diabetes, enhancing the reliability of our findings. However, limited literature during the study's preparation and resource constraints may have affected sample size estimation and follow-up duration, respectively. Consequently, we relied on past studies on ART, specifically protease inhibitors, which may have affected the accuracy of our sample estimate. Nonetheless, our study provides valuable insights into the glycemic effects of DTG-based regimens, offering crucial information for HIV programming stakeholders.

## CONCLUSIONS

Our study found hyperglycemia incidence among HIV patients on TLD, influenced by age, marital status, study site, and duration on DTG-based regimens. Integration of hyperglycemia and diabetes screening into HIV services is essential, particularly for high-risk groups such as the elderly, people with hypertension, and obese individuals before DTG initiation. Additionally, continued blood glucose monitoring for individuals already on DTG should be emphasized to enable early detection and prompt management of hyperglycemia/diabetes. Larger cohort studies and investigations into site-related factors are recommended for targeted interventions in HIV programming.

## Notes

***Acknowledgments.*** The authors extend their gratitude to the study participants, hospital administrators, staff, and healthcare workers. Their contributions at various levels were instrumental in making this study a success.

***Author contributions.*** C. An., C. Ag., Y. K., and S. Nak. conceived and designed the study. C. An., C. Ag., S. Nak., S. Nam., I. K., S. M. C., P. K., and J. E. did the data collection and interpretation. I. K. and S. Nak. participated in analysis. M. O., I. M., D. N. K., V. N., H. B. N., and B. M. participated in the interpretation of results and writing of the final manuscript. All authors read and approved the final version of the manuscript.

***Financial support.*** This study was funded the Uganda National Drug Authority.
